# Safety Monitoring of mRNA COVID-19 Vaccine Third Doses Among Children Aged 6 Months–5 Years — United States, June 17, 2022–May 7, 2023

**DOI:** 10.15585/mmwr.mm7223a2

**Published:** 2023-06-09

**Authors:** Anne M. Hause, Paige Marquez, Bicheng Zhang, Pedro L. Moro, Tanya R. Myers, Colin Bradley, Samaneh Bazel, Sarada S. Panchanathan, Tom T. Shimabukuro, David K. Shay

**Affiliations:** ^1^CDC COVID-19 Emergency Response Team; ^2^Booz Allen Hamilton, Inc., McLean, Virginia; ^3^Food and Drug Administration, Silver Spring, Maryland.

As of May 7, 2023, CDC’s Advisory Committee on Immunization Practices (ACIP) recommends that all children aged 6 months–5 years receive at least 1 age-appropriate bivalent mRNA COVID-19 vaccine dose. Depending on their COVID-19 vaccination history and history of immunocompromise, these children might also need additional doses* (*1*–*3*). Initial vaccine safety findings after primary series vaccination among children aged 6 months–5 years showed that transient local and systemic reactions were common whereas serious adverse events were rare (*4*). To characterize the safety of a third mRNA COVID-19 vaccine dose among children aged 6 months–5 years, CDC reviewed adverse events and health surveys reported to v-safe, a voluntary smartphone-based U.S. safety surveillance system established by CDC to monitor health after COVID-19 vaccination (https://vsafe.cdc.gov/en/) and the Vaccine Adverse Event Reporting System (VAERS), a U.S. passive vaccine safety surveillance system co-managed by CDC and the Food and Drug Administration (FDA) (https://vaers.hhs.gov/) (*5*). During June 17, 2022–May 7, 2023, approximately 495,576 children aged 6 months–4 years received a third dose (monovalent or bivalent) of Pfizer-BioNTech vaccine and 63,919 children aged 6 months–5 years received a third dose of Moderna vaccine.^†^ A third mRNA COVID-19 vaccination was recorded for 2,969 children in v-safe; approximately 37.7% had no reported reactions, and among those for whom reactions were reported, most reactions were mild and transient. VAERS received 536 reports after a third dose of mRNA COVID-19 vaccine for children in these age groups; 98.5% of reports were nonserious and most (78.4%) were classified as a vaccination error.^§^ No new safety concerns were identified. Preliminary safety findings after a third dose of COVID-19 vaccine for children aged 6 months–5 years are similar to those after other doses. Health care providers can counsel parents and guardians of young children that most reactions reported after vaccination with Pfizer-BioNTech or Moderna vaccine were mild and transient and that serious adverse events are rare.

Starting June 19, 2022, parents could enroll children aged 6 months–4 years in v-safe after mRNA COVID-19 vaccination.^¶^ A parent with a v-safe account could register a child or adolescent aged <16 years, receive text message reminders, and complete health surveys on behalf of the child.** Health surveys sent daily during the week after vaccination inquire about local injection site and systemic reactions and health impacts; respondents can provide additional information via free-text responses. CDC’s v-safe call center contacts registrants who report receiving medical care to request more information; registrants are encouraged to complete a VAERS report, if indicated.

VAERS accepts reports of postvaccination adverse events from health care providers, vaccine manufacturers, and members of the public.^††^ Signs, symptoms, and diagnoses reported to VAERS are assigned Medical Dictionary for Regulatory Activities (MedDRA) preferred terms (PTs) by VAERS staff members.^§§^

This report includes data for children aged 6 months–5 years who received a third mRNA COVID-19 dose during June 17, 2022–May 7, 2023.^¶¶^ Local and systemic reactions and health impacts reported during the week after vaccination were described for v-safe registrants aged 6 months–5 years. VAERS reports were described by serious and nonserious classification, demographic characteristics, and MedDRA PTs. Serious reports*** and reports not specifying vaccination error were reviewed by CDC physicians to form a consensus clinical impression based on available data. All analyses were conducted using SAS software (version 9.4; SAS Institute). These activities were reviewed by CDC and conducted consistent with applicable federal law and CDC policy.^†††^

## Review of v-safe Data

During June 17, 2022–May 7, 2023, a total of 2,969 v-safe registrants aged 6 months–5 years received a third COVID-19 vaccine dose and had at least one health survey completed; l,082 aged 6 months–2 years and 823 aged 3–4 years received Pfizer-BioNTech vaccine and 580 aged 6 months–2 years and 484 aged 3–5 years received Moderna vaccine. Concomitant receipt of another vaccine was reported for 437 (22.9%) children who received Pfizer-BioNTech and 66 (6.2%) who received Moderna; most (423; 84.1%) children received co-administered influenza vaccine.

Local and systemic reactions reported after receipt of either Pfizer-BioNTech or Moderna vaccines were most commonly reported (930; 53.2%) on the day after vaccination; 1,119 (37.7%) children had no reported reactions (Table 1). Local reactions were reported for 215 (19.9%) children aged 6 months–2 years and 304 (36.9%) aged 3–4 years after Pfizer-BioNTech vaccination and for 179 (30.9%) aged 6 months–2 years and 252 (52.1%) aged 3–5 years after Moderna vaccination. Systemic reactions were reported for 617 (57.0%) children aged 6 months–2 years and for 361 (43.9%) children aged 3–4 years after Pfizer-BioNTech vaccination, and for 309 (53.3%) children aged 6 months–2 years and for 229 (47.3%) children aged 3–5 years after Moderna vaccination. The most commonly reported reactions after receipt of either vaccine among children aged 6 months–2 years were irritability or crying, injection site pain, sleepiness, and fever (Table 2). Among children aged 3–5 years, the most frequently reported reactions were injection site pain, fatigue, and fever. Most reactions were described as mild in severity (noticeable, but not problematic).

**TABLE 1:** Reactions and health impacts reported to v-safe during days 0–7 postvaccination among children aged 6 months–5 years* who received a third dose of Pfizer-BioNTech or Moderna COVID-19 vaccine — United States, June 17, 2022–May 7, 2023

Event	No. (%) reporting reaction or health impact after vaccination,^†^ by vaccine and age group				
	Pfizer-BioNTech n = 1,905		Moderna n = 1,064		Total N = 2,969
	6 mos–2 yrs n = 1,082	3–4 yrs n = 823	6 mos–2 yrs n = 580	3–5 yrs n = 484	
**Any injection site reaction**	**215 (19.9)**	**304 (36.9)**	**179 (30.9)**	**252 (52.1)**	**950 (32.0)**
Itching	NA	28 (3.4)	NA	13 (2.7)	**41 (1.4)**
Pain	159 (14.7)	276 (33.5)	130 (22.4)	231 (47.7)	**796 (26.8)**
Redness	66 (6.1)	51 (6.2)	66 (11.4)	56 (11.6)	**239 (8.1)**
Swelling or hardness	33 (3.1)	21 (2.6)	51 (8.8)	33 (6.8)	**138 (4.7)**
Groin or underarm swelling/tenderness	2 (0.2)	NA	2 (0.3)	NA	**4 (0.1)**
**Any systemic reaction**	**617 (57.0)**	**361 (43.9)**	**309 (53.3)**	**229 (47.3)**	**1,516 (51.1)**
Abdominal pain	NA	28 (3.4)	NA	29 (6.0)	**57 (1.9)**
Myalgia	NA	47 (5.7)	NA	41 (8.5)	**88 (3.0)**
Chills	NA	40 (4.9)	NA	32 (6.6)	**72 (2.4)**
Fatigue	NA	228 (27.7)	NA	139 (28.7)	**367 (12.4)**
Fever	196 (18.1)	143 (17.4)	113 (19.5)	121 (25.0)	**573 (19.3)**
Headache	NA	50 (6.1)	NA	38 (7.9)	**88 (3.0)**
Joint pain	NA	10 (1.2)	NA	4 (0.8)	**14 (0.5)**
Nausea	NA	27 (3.3)	NA	26 (5.4)	**53 (1.8)**
Diarrhea	68 (6.3)	49 (6.0)	30 (5.2)	20 (4.1)	**167 (5.6)**
Rash	44 (4.1)	16 (1.9)	20 (3.5)	9 (1.9)	**89 (3.0)**
Vomiting	46 (4.3)	33 (4.0)	37 (6.4)	29 (6.0)	**145 (4.9)**
Irritability/Crying	438 (40.5)	NA	214 (36.9)	NA	**652 (22.0)**
Loss of appetite	151 (14.0)	NA	77 (13.3)	NA	**228 (7.7)**
Sleepiness	245 (22.6)	NA	110 (19.0)	NA	**355 (12.0)**
Other	192 (17.7)	144 (17.5)	67 (11.6)	74 (15.3)	**477 (16.1)**
**No reported reaction^§^**	**414 (38.3)**	**333 (40.5)**	**219 (37.8)**	**153 (31.6)**	**1,119 (37.7)**
**Any health impact**	**145 (13.4)**	**122 (14.8)**	**72 (12.4)**	**76 (15.7)**	**415 (14.0)**
Unable to perform normal daily activities	65 (6.0)	61 (7.4)	39 (6.7)	46 (9.5)	**211 (7.1)**
Unable to attend child care facility or school	95 (8.8)	85 (10.3)	36 (6.2)	44 (9.1)	**260 (8.8)**
Needed medical care	31 (2.9)	26 (3.2)	20 (3.5)	13 (2.7)	**90 (3.0)**
Telehealth	8 (0.7)	4 (0.5)	5 (0.9)	3 (0.6)	**20 (0.7)**
Clinic	23 (2.1)	19 (2.3)	11 (1.9)	8 (1.7)	**61 (2.1)**
Emergency department visit	1 (0.1)	4 (0.5)	2 (0.3)	0 (—)	**7 (0.2)**
Hospitalization	0 (—)	1 (0.1)	0 (—)	0 (—)	**1 (0.03)**
Other	3 (0.3)	4 (0.5)	4 (0.7)	4 (0.8)	**15 (0.5)**

**Abbreviation**: NA = not applicable.

* Data were included for children aged 6 months–4 years who received a third Pfizer-BioNTech dose during June 17, 2022–May 7, 2023, and for children aged 6 months–5 years who received a third Moderna dose during June 17, 2022–May 7, 2023.

^†^ Children whose parents reported a reaction or health impact at least once during days 0–7 postvaccination. Specific questions were included for children aged 6 months–2 years who might not be able to describe reactions or who might experience different reactions from those experienced by children aged ≥3 years.

^§^ Children whose parents or guardians reported neither injection site nor systemic reactions.

**TABLE 2:** Reactions most commonly reported to v-safe during days 0–7 postvaccination for children ages 6 months–5 years (N = 2,969)* who received a third dose of Pfizer-BioNTech or Moderna COVID-19 vaccine, by severity — United States, June 17, 2022–May 7, 2023

Reaction or health impact (age group)	No. (%) reporting reaction or health impact after vaccination^†^			
	6 mos–2 yrs n = 1,662		3–5 yrs n = 1,307	
	Pfizer-BioNTech n = 1,082	Moderna n = 580	Pfizer-BioNTech n = 823	Moderna n = 484
**Irritability/Crying (6 mos–2 yrs)/Injection site pain (3–5 yrs)**	**438 (40.5)**	**214 (36.9)**	**276 (33.5)**	**231 (47.7)**
Mild	265 (24.5)	134 (23.1)	239 (29.0)	193 (39.9)
Moderate	159 (14.7)	79 (13.6)	37 (4.5)	33 (6.8)
Severe	14 (1.3)	1 (0.2)	0 (—)	5 (1.0)
**Sleepiness (6 mos–2 yrs)/Fatigue (3–5 yrs)**	**245 (22.6)**	**110 (19.0)**	**228 (27.7)**	**139 (28.7)**
Mild	192 (17.7)	89 (15.3)	132 (16.0)	77 (15.9)
Moderate	42 (4.4)	20 (3.5)	86 (10.5)	55 (11.4)
Severe	5 (0.5)	1 (0.2)	10 (1.2)	7 (1.5)
**Fever^§^ (6 mos–5 yrs)**	**196 (18.1)**	**113 (19.5)**	**143 (17.4)**	**121 (25.0)**
Temperature not taken	63 (5.8)	27 (4.7)	33 (4.0)	29 (6.0)
Temperature taken	133 (12.3)	86 (14.8)	110 (13.4)	92 (19.0)
Normal	48 (4.4)	42 (7.2)	39 (4.7)	32 (6.6)
Fever	85 (7.9)	44 (7.6)	71 (8.6)	60 (12.4)
Mild	38 (3.6)	18 (3.1)	23 (2.8)	26 (5.4)
Moderate	29 (2.7)	9 (1.6)	33 (4.0)	16 (3.3)
Severe	15 (1.4)	13 (2.2)	12 (1.5)	16 (3.3)
Very severe	3 (0.3)	4 (0.7)	3 (0.4)	2 (0.4)
**Injection site pain (6 mos–2 yrs)/Injection site redness (3–5 yrs)**	**159 (14.7)**	**130 (22.4)**	**51 (6.2)**	**56 (11.6)**
Mild	132 (12.2)	108 (18.6)	43 (5.2)	49 (10.1)
Moderate	26 (2.4)	22 (3.8)	7 (0.9)	4 (0.8)
Severe	1 (0.1)	0 (—)	1 (0.1)	3 (0.6)
**Loss of appetite (6 mos–2 yrs)/Myalgia (3–5 yrs)**	**151 (14.0)**	**77 (13.3)**	**47 (5.7)**	**41 (8.5)**
Mild	96 (8.9)	46 (7.9)	21 (2.6)	21 (4.33)
Moderate	44 (4.1)	27 (4.7)	25 (3.0)	15 (3.1)
Severe	11 (1.0)	4 (0.7)	1 (0.1)	5 (1.0)

* Data were included for children aged 6 months–4 years who received a third Pfizer-BioNTech dose during June 17, 2022–May 7, 2023, and for children aged 6 months–5 years who received a third Moderna dose during June 17, 2022–May 7, 2023.

**^†^** Children whose parents or guardians reported a reaction or health impact at least once during days 0–7 after vaccination. Includes the most severe event reported during the 0–7 window. Parents and guardians who participate in v-safe use the following definitions to describe the severity of a child’s symptoms: mild (noticeable, but not problematic), moderate (limit normal daily activities), or severe (make daily activities difficult or impossible).

^§^ Fever is self-reported and registrants are not required to record a temperature. Among children who had a reported temperature and met the definition for fever (temperature ≥100.4°F [≥38.0°C]) during days 0–3, fever was classified as mild (100.4–101.1°F [38.0–38.4°C]), moderate (101.2–102.0°F [38.4–38.9°C]), severe (102.1–104.0°F [38.9–40.0°C]), or very severe (>104.0°F [>40.0°C]).

Parents of 211 (7.1%) children aged 6 months–5 years reported at least once during the week after vaccination that their child was unable to perform normal daily activities, including 126 Pfizer BioNTech recipients and 85 Moderna recipients (Table 1); 90 (3.0%) parents reported seeking medical care for their child. Among these, 61 (67.8%) reported that care was received in an outpatient clinic; one child was hospitalized for pneumonia; 43 (48.0%) had additional information available from the v-safe call center. Parents of 30 children indicated that medical care was unrelated to vaccination, 11 parents completed a VAERS report, and two parents indicated that the report was made in error. Symptoms or signs of infection (e.g., cough, conjunctivitis, or hand-foot-and-mouth disease rash) were reported via free text or VAERS report for 34 of the 90 reports of medical care.

## Review of VAERS Data

During June 17, 2022–May 7, 2023, VAERS received and processed 407 reports of adverse events among children aged 6 months–4 years after receipt of Pfizer-BioNTech vaccine and 129 reports for children aged 6 months–5 years after Moderna vaccine (Table 3).^§§§^ Most reports were for children who received COVID-19 vaccine without concomitantly administered vaccines (487; 90.9%).

**TABLE 3:** Reports of nonserious and serious events to the Vaccine Adverse Event Reporting System for children aged 6 months–5 years* after receipt of dose 3 Pfizer-BioNTech or Moderna COVID-19 vaccine^†^ — United States, June 17, 2022–May 7, 2023

Adverse events	No. (%) reporting by vaccine		
	Pfizer-BioNTech n = 407	Moderna n = 129	Total N = 536
**Nonserious reports, total**	**401 (98.5)**	**127 (98.5)**	**528 (98.5)**
**Reports of vaccination error^§^**	**330 (81.1)**	**90 (70.9)**	**420 (78.4)**
Error without adverse health event	305 (92.4)	88 (97.8)	**393 (93.6)**
Error with adverse health event^¶^	25 (7.6)	2 (2.2)	**27 (6.4)**
Reports not specifying vaccination error**	**71 (17.4)**	**37 (29.1)**	**108 (20.4)**
Diarrhea/Vomiting	6 (8.5)	1 (2.7)	**7 (6.5)**
Febrile seizure	2 (2.8)	0 (—)	**2 (1.9)**
Fever	2 (2.8)	3 (8.1)	**5 (4.6)**
Hives	2 (2.8)	6 (16.2)	**8 (7.4)**
Infection, COVID-19	5 (7.0)	7 (18.9)	**12 (11.1)**
Infection, other	31 (43.7)	14 (37.8)	**45 (41.7)**
Injection site reaction	1 (1.4)	0 (—)	**1 (0.9)**
No adverse event	11 (15.5)	2 (5.4)	**13 (12.0)**
Rash	6 (8.5)	3 (8.1)	**9 (8.3)**
Other	5 (7.0)	1 (2.7)	**6 (5.6)**
**Serious reports, total^††, §§^**	**6 (1.5)**	**2 (1.6)**	**8 (1.5)**

**Abbreviations**: MedDRA PT = Medical Dictionary for Regulatory Activities preferred term; VAERS = Vaccine Adverse Event Reporting System.

* Signs and symptoms in VAERS reports are assigned MedDRA PTs by VAERS staff members. Each VAERS report might be assigned more than one MedDRA PT, which can include normal diagnostic findings. A MedDRA PT does not indicate a medically confirmed diagnosis.

^†^ Reports to VAERS were included for children aged 6 months–4 years who received a third monovalent Pfizer-BioNTech dose during June 17, 2022–May 7, 2023, or a bivalent Pfizer-BioNTech dose during December 8, 2022–May 7, 2023, and for children aged 6 months–5 years who received a third monovalent Moderna dose during June 17, 2022–May 7, 2023, or a bivalent Moderna dose during December 8, 2022–May 7, 2023.

^§^ The most common vaccination error MedDRA PTs among reports of vaccination error included incorrect product formulation administered (112; 26.7%), inappropriate schedule of product administration (86; 20.5%), expired product administered (47; 11.2%), and incorrect dose administered (43; 10.2%).

^¶^ The most common adverse health event MedDRA PTs for reports with nonserious vaccination errors included fever (seven; 25.9%), pain in extremity (four; 14.8%), and chills (three; 11.1%).

** Nonserious reports not specifying vaccination error were reviewed by CDC physicians to form a clinical impression and are listed in the table.

^††^ VAERS reports are classified as serious if any of the following are reported: hospitalization, prolongation of hospitalization, life-threatening illness, permanent disability, congenital anomaly or birth defect, or death. https://www.meddra.org/how-to-use/basics/hierarchy

^§§^ Serious reports to VAERS were reviewed by CDC physicians to form a clinical impression. Clinical impressions included acute hemorrhagic edema of infancy (with human coronavirus OC43 infection), diabetic ketoacidosis (following new onset diabetes mellitus type 1), Henoch-Schönlein purpura, Kawasaki disease, new onset afebrile seizure (two), pneumonia, and viral exacerbation of asthma. After review of all available information, no evidence suggested that these reported events were related to vaccination.

The most common events reported (420; 78.4%) were vaccination errors (e.g., incorrect product formulation administered, inappropriate schedule of product administration, expired product administered, or incorrect dose administered). Among 330 reports of vaccination errors after administration of Pfizer-BioNTech and 90 after Moderna vaccines, 27 (5.0%) reports indicated that an adverse health event had occurred.

After the exclusion of reports specifying vaccination error, 108 nonserious reports remained, including 71 after Pfizer-BioNTech and 37 after Moderna vaccination. After review, the most commonly reported nonserious events included infection^¶¶¶^ (57; 52.8%), no adverse event (13; 12.0%), and rash (nine; 8.3%).

Eight (1.5%) serious reports were made to VAERS: six for children who received Pfizer-BioNTech and two for children who received Moderna. Clinical impressions of the serious reports included acute hemorrhagic edema of infancy (infection with non–SARS-CoV-2 coronavirus), diabetic ketoacidosis (following new onset of diabetes mellitus type 1), Henoch-Schönlein purpura, Kawasaki disease, new-onset afebrile seizure (two), pneumonia, and viral exacerbation of asthma. After review of all available information, no evidence suggested that these reported events were related to vaccination.

## Discussion

This report provides findings from v-safe and VAERS for children aged 6 months–5 years who received a third dose of mRNA COVID-19 vaccine during June 17, 2022–May 7, 2023; during this period, approximately 559,495 third doses were administered to children in this age group. The findings in this report are consistent with those from postauthorization safety surveillance of the first 2 doses of mRNA COVID-19 vaccines. Systemic reactions after receipt of the first or second doses were more commonly reported for children aged 6 months–2 years than for those aged 3–5 years; 44.7% of VAERS reports included at least one vaccination error (*4*).

Reports to v-safe of injection site and systemic reactions after a third dose of mRNA COVID-19 vaccine were similar in frequency to those reported after a first (19.0%–32.4% reports of injection reaction and 32.2%–55.8% reports of systemic reaction) or second dose (18.3%–47.1% reports of injection reaction and 29.2%–58.2% reports of systemic reaction) among children aged 6 months–5 years (*4*). They were less frequent than those reported for children aged 5–11 years after a monovalent or bivalent booster dose (*6*,*7*). Approximately 38% of parents reported to v-safe that their child experienced no reactions in the week after a third mRNA vaccine dose. Most parents of children who received medical care reported that care was unrelated to vaccination. Many children who received medical care had signs and symptoms of an acute infection.

After administration of approximately 550,000 third doses of mRNA COVID-19 vaccine to children aged 6 months–5 years, eight serious reports were received by VAERS. More than 98.0% of reports were nonserious; most represented vaccination errors (78.4%). Vaccination errors have constituted a large proportion of VAERS reports among children. For example, 85.0% of VAERS reports for children aged 5–11 years after bivalent booster vaccination were related to vaccination error (*7*). Among reports not specifying vaccination error, one half were of an unrelated infection. Recent ACIP recommendations simplifying guidance for bivalent vaccination might reduce reports of vaccination error (*3*).

The findings in this report are subject to at least four limitations. First, v-safe is a voluntary program; as a result, data might not be representative of the vaccinated population. Second, VAERS is a passive reporting system and is subject to reporting biases and underreporting, especially of nonserious events (*5*). Third, this report combined data for monovalent and bivalent mRNA COVID-19 vaccine formulations. However, findings among older children did not differ by formulation (*7*). Finally, interpretation of these data is limited by the short surveillance period and small denominator of vaccinated children aged 6 months–5 years.

All children aged 6 months–5 years are recommended to receive at least 1 bivalent dose and might need multiple COVID-19 vaccine doses depending on their age and COVID-19 vaccination history**** (*3*). No new safety findings were identified among children aged 6 months–5 years after receipt of a third dose. Most reported reactions after vaccination were mild and transient. Although SARS-CoV-2 infection among young children typically results in mild infection, it can result in serious illness, including multisystem inflammatory syndrome in children, long-term sequalae, and death (*8*). mRNA COVID-19 vaccination provides protection against symptomatic SARS-CoV-2 infection for at least 4 months after vaccination among children aged 3–5 years (*9*). CDC and FDA will continue to monitor vaccine safety and will provide updates to help guide COVID-19 vaccination recommendations.

**Summary**

**What is already known about this topic?**

All children aged 6 months–5 years are recommended to receive ≥1 bivalent mRNA COVID-19 vaccine dose; approximately 550,000 children in these age groups have received a third monovalent or bivalent mRNA vaccine dose.

**What is added by this report?**

In v-safe, 38% of children had no reported reactions after a third dose; most reported reactions were mild and transient. Vaccination errors accounted for 78% of events reported to the Vaccine Adverse Event Reporting System.

**What are the implications for public health practice?**

Findings after receipt of a third mRNA vaccine dose among young children were similar to those described after receipt of 1 and 2 doses; no new safety concerns were identified.

## Footnotes

* On June 17, 2022, the Food and Drug Administration (FDA) amended the Emergency Use Authorizations (EUAs) for COVID-19 mRNA vaccines to include monovalent (mRNA encoding the spike protein from the SARS-CoV-2 ancestral strain) formulations of Pfizer-BioNTech (administered as 3 doses for children aged 6 months–4 years) and Moderna vaccines (administered as 2 doses for children aged 6 months–5 years) for younger children. On December 8, 2022, FDA amended EUAs for both manufacturers to include bivalent (mRNA encoding the spike protein from the SARS-CoV-2 ancestral strain and BA.4/BA.5 Omicron variants) formulations for both age groups. EUAs for both manufacturers have since been amended regarding bivalent doses; ACIP guidance has changed to reflect these updates.

† https://covid.cdc.gov/covid-data-tracker/#vaccination-demographic (Accessed April 26, 2023).

§ Vaccination errors are categorized into the following groups: administration errors, contraindication to vaccination, equipment, general, inappropriate schedule of drug administration, incorrect dose, prescribing and dispensing, product quality, product labeling/packaging, and wrong product. Section 4.5 vaccination error groups and MedDRA PTs for COVID-19 vaccination errors are available online. https://www.cdc.gov/vaccinesafety/pdf/VAERS-COVID19-SOP-02-02-2022-508.pdf

¶ On May 19, 2023, CDC closed enrollment in v-safe for COVID-19 vaccines. Health check-ins (and follow-up calls, if needed) for any doses added before May 19 will continue until June 30, 2023.

** Health check-ins are sent via text messages that link to web-based surveys on days 0–7 after vaccination; then weekly through 6 weeks after vaccination; and then 3, 6, and 12 months after vaccination. Specific questions were included for children aged 6 months–2 years who might not be able to describe reactions or who might experience reactions that differ from those experienced by children aged ≥3 years. Parents use the following definitions to describe the severity of a child’s symptoms: mild (noticeable, but not problematic), moderate (limit normal daily activities), or severe (make daily activities difficult or impossible). V-safe also sends text-message reminders when a person is eligible for their next vaccine dose; an additional reminder was sent on March 21, 2023, to parents of children who were eligible for a booster dose but had not reported one.

†† Under EUA regulations, health care providers are required to report certain adverse events after COVID-19 vaccination to VAERS, including vaccination errors and death (https://vaers.hhs.gov/faq.html). VAERS forms ask for patient, vaccine, administration, and adverse event information. https://vaers.hhs.gov/docs/VAERS%202.0_Checklist.pdf

§§ Each VAERS report might be assigned more than one MedDRA PT. A MedDRA-coded event does not indicate a medically confirmed diagnosis. https://www.meddra.org/how-to-use/basics/hierarchy

¶¶ Data for v-safe were included for children aged 6 months–4 years who received a third Pfizer-BioNTech dose during June 17, 2022–May 7, 2023, and for children aged 6 months–5 years who received a third Moderna dose during June 17, 2022–February 26, 2023. Reports to VAERS were included for children aged 6 months–4 years who received a third monovalent Pfizer-BioNTech dose during June 17, 2022–May 7, 2023, or bivalent Pfizer-BioNTech dose during December 8, 2022–May 7, 2023, and for children aged 6 months–5 years who received a third monovalent Moderna dose during June 17, 2022–May 7, 2023, or bivalent Moderna dose during December 8, 2022–May 7, 2023.

*** VAERS reports are classified as serious (based on FDA C.F.R. Title 21) if any of the following are reported: hospitalization, prolongation of hospitalization, life-threatening illness, permanent disability, congenital anomaly or birth defect, or death. https://www.accessdata.fda.gov/scripts/cdrh/cfdocs/cfcfr/cfrsearch.cfm?fr

††† 45 C.F.R. part 46, 21 C.F.R. part 56; 42 U.S.C. Sect. 241(d); 5 U.S.C. Sect. 552a; 44 U.S.C. Sect. 3501 et seq.

§§§ Processed VAERS reports are those that have been coded using MedDRA, deduplicated, and undergone standard quality assurance and quality control review.

¶¶¶ Clinical review identified 57 reports of infection: cough or wheezing (three), COVID-19 (12), croup (three), ear infection (15), influenza (one), norovirus (one), pneumonia (one), respiratory tract infection (15), hand, foot, and mouth (one), strep throat (four), and viral rash (one).

**** As of May 7, 2023, monovalent COVID-19 vaccines are no longer authorized for use in the United States.
